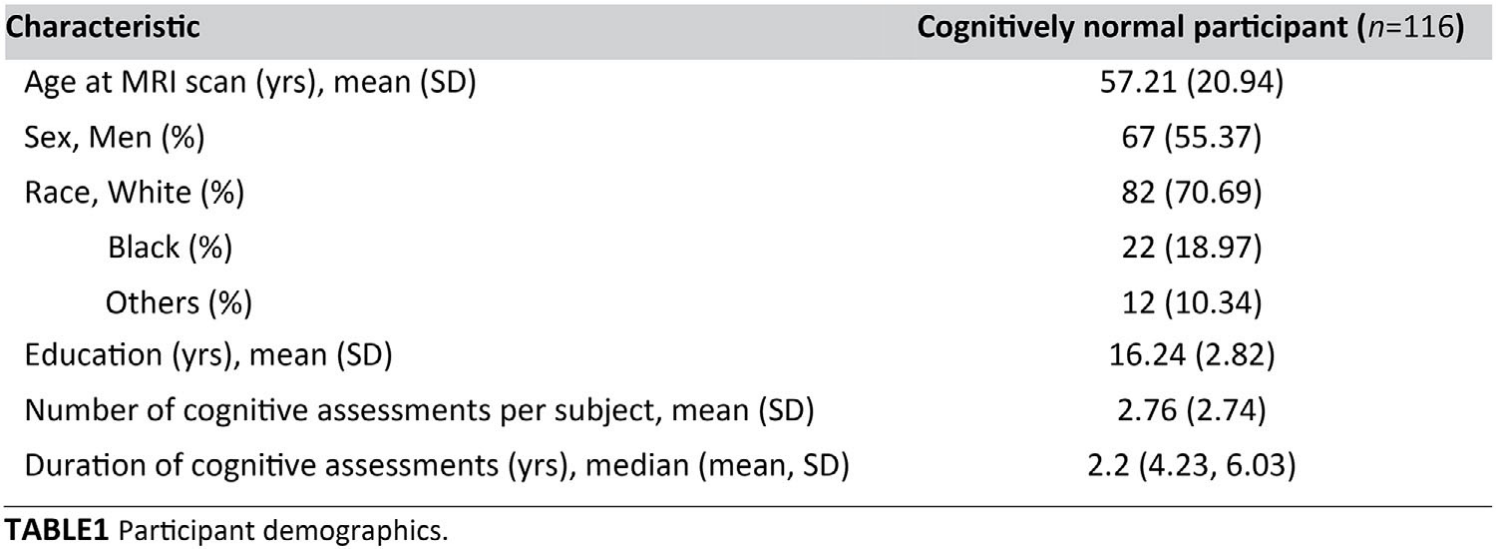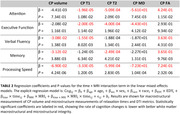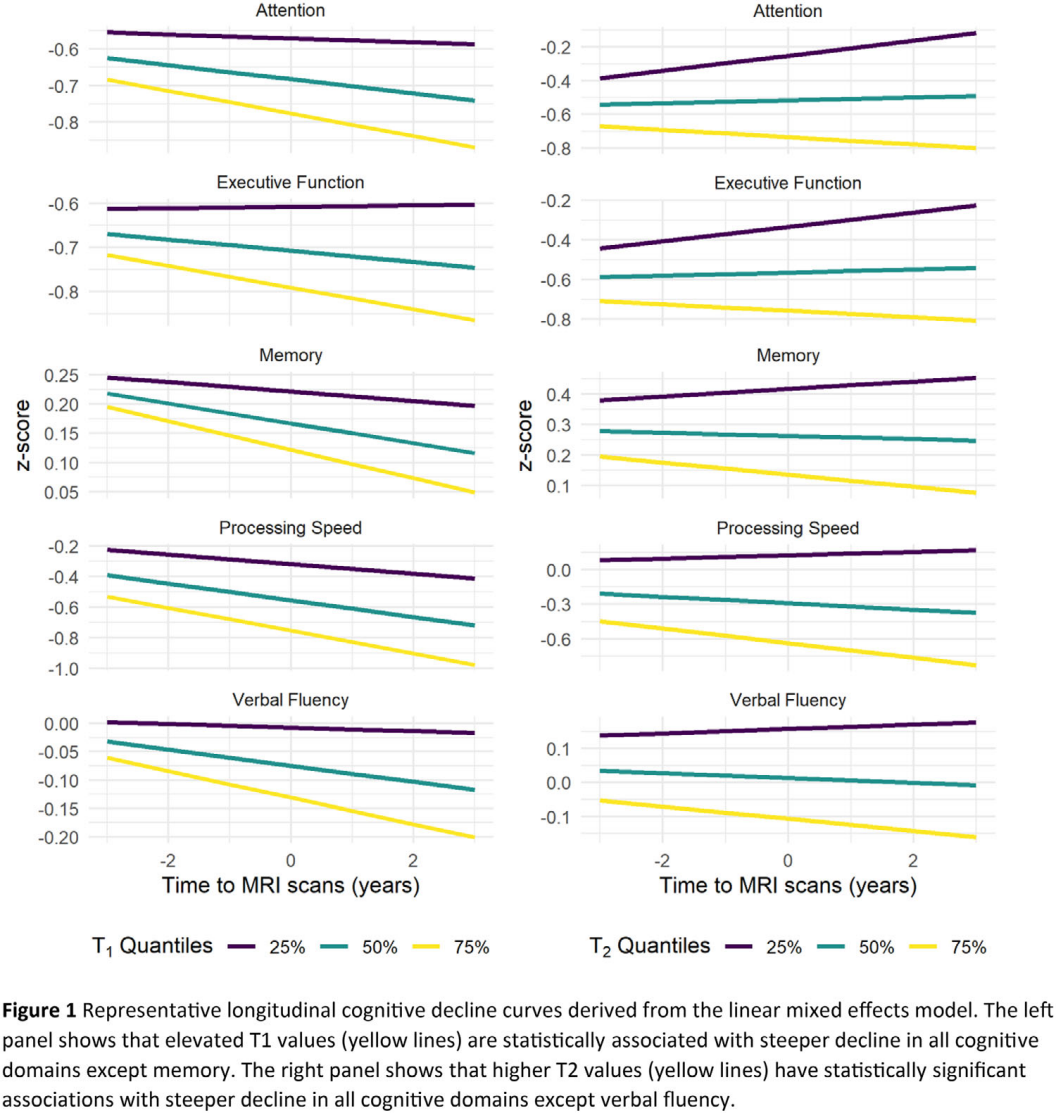# Associations Between Choroid Plexus Integrity and Cognitive Decline in Aging: Insights from Advanced MRI Analysis

**DOI:** 10.1002/alz.093777

**Published:** 2025-01-09

**Authors:** Zhaoyuan Gong, Murat Bilgel, Mary E Faulkner, Jonghyun Bae, John P Laporte, Alex Guo, Susan M. Resnick, Josephine M. Egan, Mustapha Bouhrara

**Affiliations:** ^1^ National Institute on Aging, Baltimore, MD USA; ^2^ National Institute on Aging, National Institutes of Health, Baltimore, MD USA; ^3^ Laboratory of Clinical Investigation, National Institute on Aging, Intramural Research Program, Baltimore, MD USA

## Abstract

**Background:**

The choroid plexus (CP), a vital component in the brain’s ventricles, is crucial for cerebrospinal fluid (CSF) production and maintenance of the brain’s physiological environment. It plays a key role in regulating neuroinflammatory responses, clearing harmful substances, producing neurotrophic factors and signaling molecules, and forming blood‐CSF barrier. Consequently, changes to the CP’s structural integrity could disrupt brain homeostasis and lead to cognitive impairment. Indeed, recent research has highlighted CP alterations in aging as well as in various cognitive disorders including Alzheimer’s and Parkinson’s diseases. This study investigates the correlation between the macrostructural and microstructural integrity of the CP and longitudinal cognitive changes in cognitively unimpaired individuals.

**Method:**

116 cognitively unimpaired participants from the BLSA and GESTALT underwent advanced MRI, including relaxometry (T1 and T2) and diffusion tensor imaging (DTI), enabling the quantification of CP volume, fractional anisotropy (FA), and mean diffusivity (MD). Participants received cognitive assessments at visits preceding and concurrent with MRI scans (320 visits total) to measure memory, attention, executive function, verbal fluency, and processing speed (Table 1). Linear mixed‐effects models were used to analyze the association between MRI metrics and longitudinal cognitive changes, adjusting for age, sex, education, and race.

**Result:**

Our study found that larger CP volume, elevated T1 and T2 values, and higher MD values, all indicative of compromised macrostructural or microstructural integrity, were associated with faster declines in various cognitive domains (Table 2). Particularly, the association was found in memory, verbal fluency, and processing speed for CP volume; in attention, executive function, and processing speed for T1 and T2 (Figure 1); and in attention, executive function, and processing speed for MD. Conversely, lower FA values, representing deteriorated microstructural integrity, correlated with accelerated decline in memory, attention, verbal fluency, and processing speed.

**Conclusion:**

This study highlights a significant association between CP characteristics and cognitive decline in cognitively unimpaired individuals. These findings underscore the importance of the CP in cognitive health and emphasize the need for further research into the role of the CP in healthy aging, potentially opening new pathways for early detection and intervention in cognitive decline.